# Dentoskeletal effects of early class III treatment protocol based on timing of intervention in children

**DOI:** 10.1186/s40510-021-00392-2

**Published:** 2021-12-22

**Authors:** Ludovica Nucci, Caterina Costanzo, Marco Carfora, Fabrizia d’Apuzzo, Lorenzo Franchi, Letizia Perillo

**Affiliations:** 1 Multidisciplinary Department of Medical-Surgical and Dental Specialties, University of Campania Luigi Vanvitelli, Via L. De Crecchio 6, 80138 Naples, Italy; 2 Department of Experimental and Clinical Medicine, University of Florence, Florence, Italy

**Keywords:** Early treatment, Timing, Class III dentoskeletal malocclusion, Cervical vertebral maturation stage, Modified SEC III protocol, Mixed dentition

## Abstract

**Background:**

To detect the optimal timing of intervention based on different cervical vertebral maturation stage (CS1-2 vs. CS3-4) for the treatment of Class III malocclusion with early Class III protocol.

**Methods:**

A total sample of 43 patients (23 females, 20 males) ranging between 7 and 13 years of age with dentoskeletal Class III malocclusion treated with the modified SEC III (Splints, Elastic and Chincup) protocol divided into two groups based on the cervical vertebral maturation stages (CS1-2 and CS3-4) was included in this retrospective observational longitudinal study. Patient compliance was assessed using a 2-point Likert scale. Statistical comparisons between the two groups were performed with independent sample *t* tests.

**Results:**

No statistically significant differences for any of the cephalometric variables describing the baseline dentoskeletal features were found between the two groups except for the mandibular unit length that was significantly greater in the pubertal group (*P* = 0.005). The modified SEC III protocol produced favorable sagittal outcomes in both groups, whereas no statistically significant T1-T2 changes were found between the CS1-2 and CS3-4 groups for any of the angular and linear measurements. No significant differences were found in the prevalence rates of the degree of collaboration between the two groups (*P* = 1.000).

**Conclusions:**

No significant differences between prepubertal and pubertal patients were found in the sagittal and vertical dentoskeletal changes with the modified SEC III protocol. Thus, this early Class III treatment produced similar favorable effects in growing subjects regardless of the cervical vertebral maturation stages from CS1 to CS4.

## Background

The optimal timing to start an orthodontic treatment evaluating the individual skeletal maturation is always a debated topic [1–3]. However, an early approach in patients with dentoskeletal Class III malocclusion is mostly accepted to intercept the environmental factors involved in the manifold etiology of the different clinical patterns [4–6] based on different interactions among them and genetic/epigenetic factors [7, 8]. Several authors prefer an early treatment approach, as soon as the diagnosis is formulated, to harmonize the maxillary and mandibular growth controlling, as much as possible, the worsening factors of Class III disharmonies [9, 10]. Moreover, orthopedic therapy leads to shorter fixed treatment in permanent dentition and eventual less invasive surgery at the end of mandibular growth [3, 11].

Moreover, several studies showed the high impact of the malocclusion on personal psychological well-being [12, 13]. In this context, early Class III treatment should be considered as the best choice to avoid or reduce any social problems of children during their growth, e.g. bullying, depression, and others [14].

The SEC III treatment protocol (Splints, Class III Elastics and Chincup) was conceived to perform skeletal and occlusal correction of Class III malocclusion reducing incisor compensation while controlling the vertical intermaxillary dimension [15–17]. Lately, a modified SEC III protocol was described and included a bonded maxillary expander, instead of the upper acrylic splint, to correct the transversal maxillary constriction and/or crowding as well as to control the clockwise mandibular rotation [18–20]. Even though one of the main cons of modified SEC III protocols is the need of patient compliance wearing both splints, elastics, and chincup, previous studies showed that patients usually have good to moderate collaboration and even when the compliance with the extraoral appliance is poor, there are still favorable effects on the sagittal dentoskeletal components [19, 21, 22].

Timing for Class III orthopedic treatment is a sticking point in planning the interceptive therapy to get successful outcomes by the end of pubertal stage. It was shown that early interventions favor better skeletal results, whereas late approaches reduce the skeletal effects causing greater dental compensation [6, 23, 24].

Thus, the purpose of this study was to detect the optimal timing for the treatment of Class III malocclusion with one of the protocols used in growing patients, the modified SEC III protocol, dividing them by cervical vertebral maturation stage (CS) (CS1-2 vs. CS3-4).

## Methods

The study was performed in accordance with the Declaration of Helsinki and approved by the Ethics Committee of the University of Campania *Luigi Vanvitelli* (Prot. N° 222/2018). An informed consent for the use of personal data was obtained from parents of each patient. This retrospective observational longitudinal study collected records of patients ranging between 7 and 13 years of age with dentoskeletal Class III malocclusion consecutively treated between 2015 and 2019 with the modified SEC III protocol at the Orthodontic Program of the University of Campania *Luigi Vanvitelli,* Naples (Italy).

The sample size was computed considering *α* = 0.05, power = 0.80, an effect size of 1 calculated from the standard deviation of 1.5° for the variable ANB derived from a previous study [22]. A sample size of 34 patients (at least 17 patients for each group) was determined to be adequate.

Inclusion criteria for the study were:

- high quality initial and post-treatment radiographs;
- Class III dentoskeletal malocclusion;
- anterior crossbite or edge-to-edge incisor relationship;
- Wits appraisal ≤ 0 mm;
- cervical vertebral maturation stage [25–27] before treatment between CS1-4.

Exclusion criteria were congenital anomalies or craniofacial syndromes; sequelae of traumatic injuries; previous orthopedic/orthodontic treatment; patients for whom no collaboration with the removable appliances was reported.

To note, it was not possible to exclude patients with pseudo-Class III due to the difficulties in differentiating between pseudo-Class III and Class III in a retrospective sample with no possibility to perform any clinical evaluation. For each subject, the diagnostic records were analyzed before treatment (T1) and at the end of the orthopedic phase (T2). A total sample of 43 patients was included (23 females and 20 males). Patients were divided into two different groups based on the different skeletal maturation at the start of treatment: the first group had CS1-2 while the second group presented with CS3-4.

### Treatment protocol

**Fig. 1:**
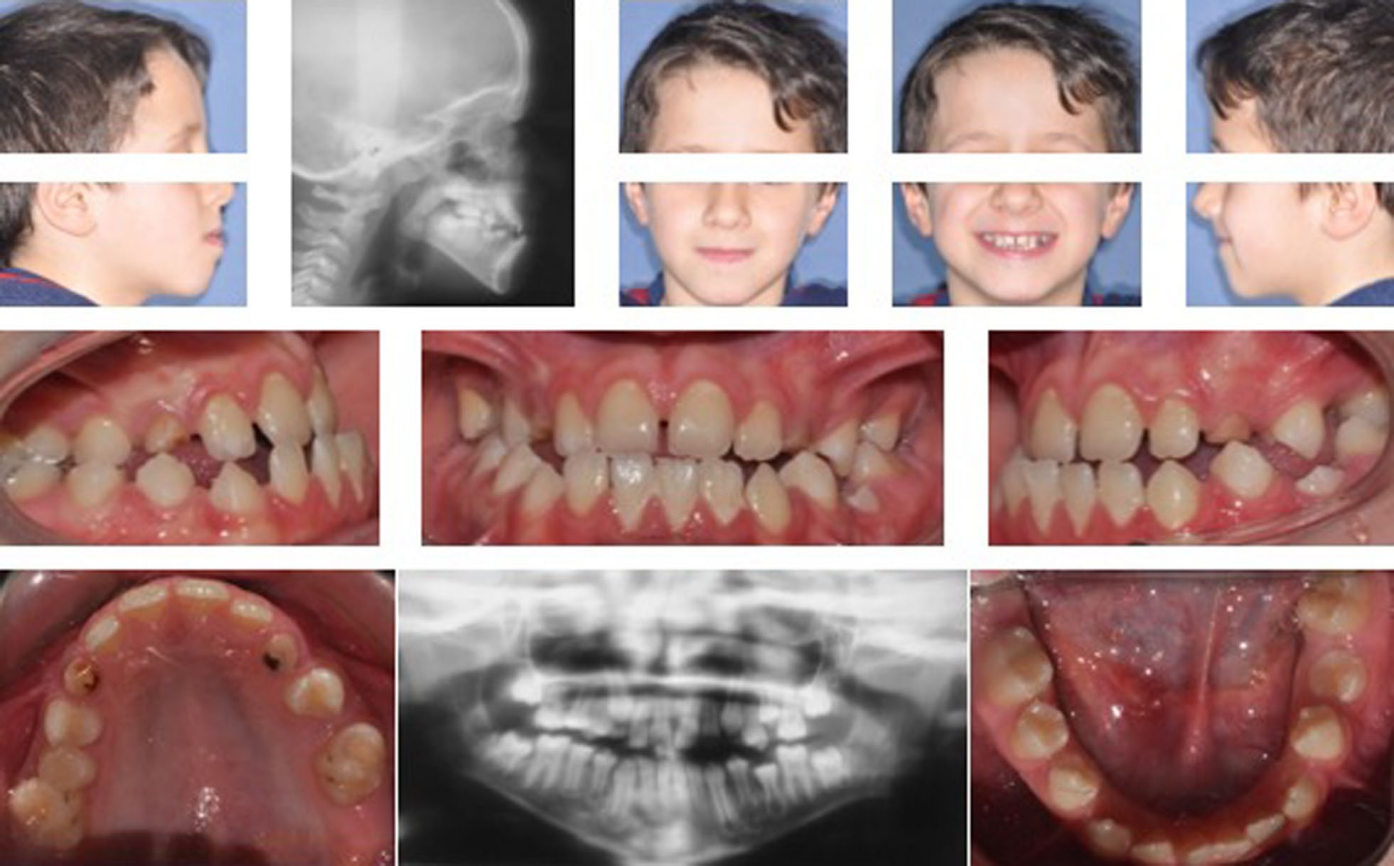
Pretreatment records of patient with Class III malocclusion in CS2 (at T1)

Treatment with the modified SEC III protocol was described in detail in previous studies [18, 19, 22] and it was applied to patients in the different CS (Fig. 1).

**Fig. 2:**
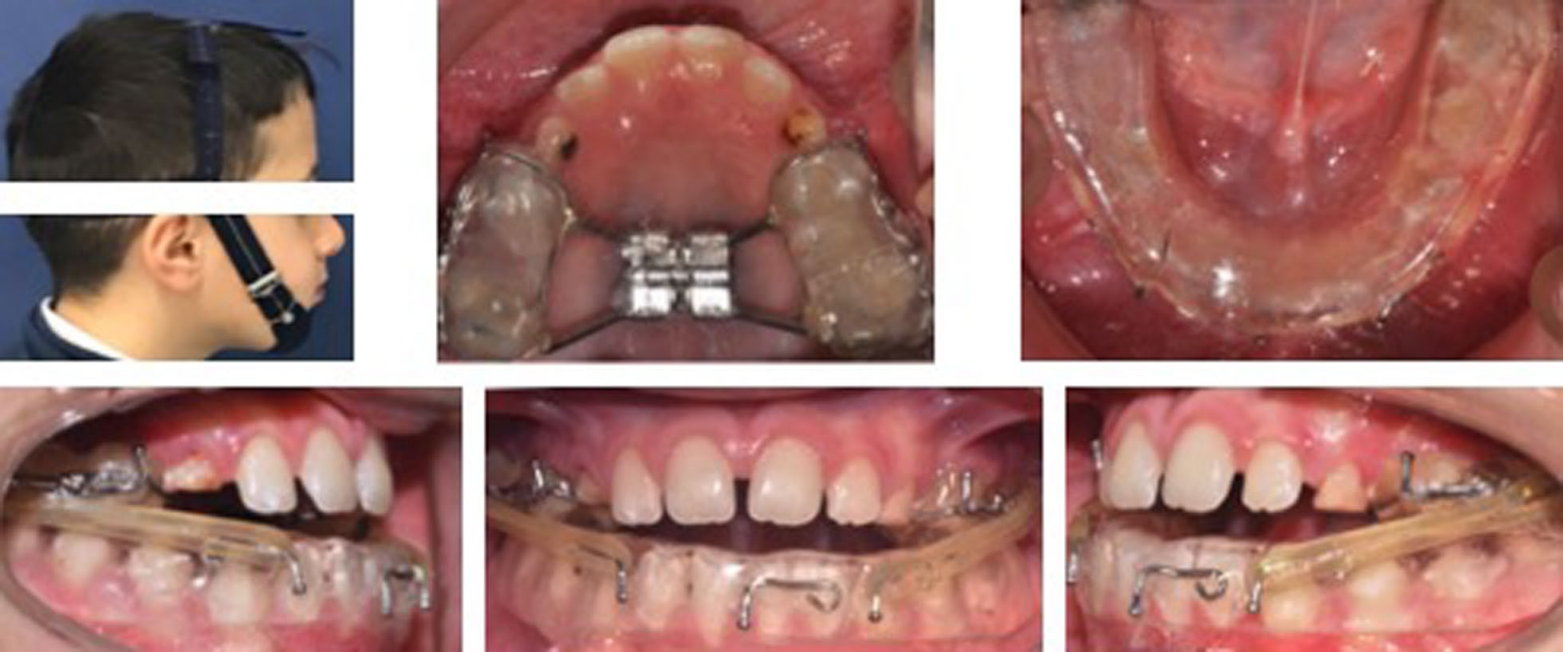
Photographs during treatment with modified SEC III protocol

Briefly, the modified SEC III protocol is composed by an upper bonded maxillary expander applied on the deciduous canines and the first and second deciduous molars or also on the permanent first molars when erupted, and a lower removable splint in acrylic resin extended from the last molars bilaterally (Fig. 2). On the lower splint a single contact point with the bonded expander at the level of the last molars [19].

The anterior elastic (force between 225 and 425 g per side) was applied on two hooks placed on the bonded expander in the canine area and one in midline area of the lower splint, while the bilateral Class III elastics were placed on two hooks bilaterally one embedded in the molar area of the upper bonded expander and one in the canine area of the lower splint. The force of the elastics was chosen differently in each case considering patient’s overbite and toleration to them. The extraoral chincup was applied with force vector passing through the upper first molars area to avoid extrusion of the posterior teeth and consequent clockwise mandibular rotation with a force ranged from 400 to 600 g [18, 19, 22]. The expander was activated at the chairside with two turns of the expansion screw by the orthodontist, then the activation continued at home with two turns per day until the palatal cusps of the upper molars approximated the buccal cusps of the lower molars. The patients were weekly monitored, and the expansion phase lasted around 1–3 weeks depending on the degree of maxillary constriction and/or crowding [28–30].

Scaling and professional cleaning was planned every three months to maintain an appropriate oral hygiene during treatment.

**Fig. 3:**
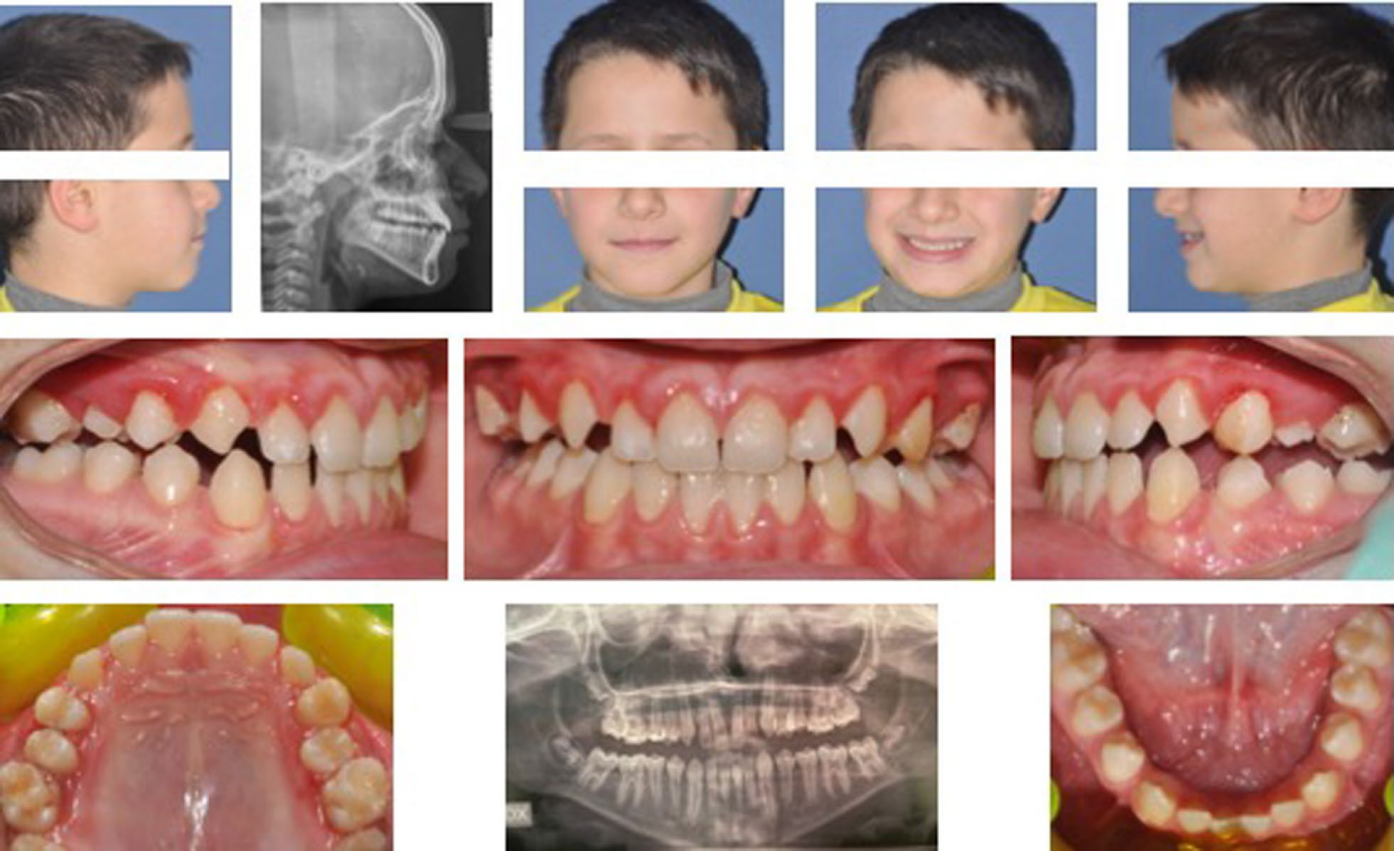
Records at the end of treatment with modified SEC III protocol (at T2)

Patients were asked for a strong collaboration with the removable devices and auxiliaries: lower splint and elastics for a minimum of 16 h per day and chincup 14 h per day. The treatment with the modified SEC III lasted until a positive overjet of at least 2 mm was obtained (Fig. 3).

### Compliance appraisal

Information on patient compliance, reported in the clinical chart, was assessed using a 2-point Likert scale (moderate and good) and was obtained from the clinical chart after interviewing each patient and also his/her parents at each appointment to limit the overestimation of duration of wear declared by young patients [19, 22, 31].

Moderate compliance was reported when the elastics were worn between 12 and 16 h and the chincup between 10 and 12 h per day. Patients wearing removable appliances as suggested by the treatment protocol had good compliance.

### Cephalometric analysis

A customized scanning procedure and cephalometric analysis were applied to the collected cephalograms at T1 and T2 (Viewbox 4.0. Cephalometric dHAL Software, Greece). Twelve angular and two linear variables were generated for each tracing, both for T1 and T2 comparison. The enlargement factor was standardized to 0% (life-size).

### Statistical analysis

Chi-square tests were used to assess differences in gender distribution and in the degree of collaboration between the two treated groups. All cephalometric data at T1 at T2, and the T1-T2 changes were tested for normal distribution (Kolmogorov–Smirnov test). Comparisons between the two groups CS1-2 versus CS3-4 treated with modified SEC III on the dentoskeletal features at T1 (baseline characteristics), at T2, and on the T1-T2 changes were performed with either the independent sample t tests or with the Mann–Whitney U test when data were not normally distributed (Statistical Package for the Social Sciences, SPSS, Version 24, IBM, Armonk, NY).

### Method error

Fifteen lateral cephalograms, selected randomly, were traced and measured at two times after 15 days by the same operator. The error was calculated with the “method of moments” variance estimator (MME) [32]. The intraobserver reproducibility was assessed with the intraclass correlation coefficients (ICC). Intra-observer agreement for the cervical vertebral staging using the CVM method was also assessed on 15 lateral cephalograms evaluated at two times after 15 days by the same operator with the weighted kappa.

## Results

The total sample of 43 patients at the baseline was divided in two groups: the group CS1-2 was composed by 25 patients (12 females and 13 males, mean age: 8.3 years; SD: 1.1 years) and the group CS3-4 by 18 patients (11 females and 7 males, mean age: 11.5 years; SD: 1.5 years). No statistically significant differences were found in gender distribution (*P* = 0.537).

At T2, the patients in the CS1-2 group had a mean age of 9.5 years (SD: 1.1 years) while the patients in CS3-4 showed a mean age of 12.7 (SD: 1.6 years). The mean duration of treatment (T1-T2) in the two groups was 1.2 years (SD: 0.3 years) and 1.1 years (SD: 0.3 years), respectively (*P* = 0.283) (Table 1).

**Table 1:** Demographics of the prepubertal and pubertal groups

Variables	Prepubertal group (*n* = 25, 12 f, 13 m)		Pubertal group (*n* = 18, 11 f, 7 m)		*P* value
	Mean	SD	Mean	SD	
Age T1, year	8.3	1.1	11.5	1.5	0.000*
Age T2, year	9.5	1.1	12.7	1.6	0.000*
T2-T1, year	1.2	0.3	1.1	0.3	0.283
CVM stages at T1	15 CS1 11 CS2		12 CS3 6 CS4		
CVM stages at T2	7 CS1 11 CS2 7 CS3		8 CS3 10 CS4		

The bold indicates the statistical significance

*P < 0.05; *SD* standard deviations

At T1, in the prepubertal group, 15 patients were in CS1 and 11 patients in CS2, whereas 12 patients were in CS3 and 6 patients in CS4 in the pubertal group. After modified SEC III protocol (T2), in the prepubertal group 7 patients were still in CS1, 11 in CS2 and 7 in CS3, whereas in the pubertal group 8 patients remained in CS3 and 10 patients showed a CS4 (Table 1).

As for the compliance reported on the clinical chart of each patient, the first group CS1-2, 16 patients reported a good degree and 9 patients a moderate degree of collaboration with chincup, Class III, and anterior elastics. In the second group CS3-4, 12 patients had good collaboration and 6 patients a moderate degree of collaboration with the abovementioned removable appliances. No significant differences were found in the prevalence rates of the degree of collaboration between the two groups (Fisher’s exact probability test (*P* = 1.000).

The inter-rater agreement to assess the CS showed a weighted kappa of 0.96. The values for the MME for the angular measurements varied from 0.2 degrees to 0.3 degrees and for the linear measurements varied from 0.2 mm to 0.3 mm (Table 2). As for the values of ICC they were all above 0.99 indicating very good intraobserver reproducibility (Table 2).

**Table 2:** Values for the random error assessed with the method of moments’ estimator (MME) of the Springate, and intraclass correlation coefficient (ICC)

Variables	MME	ICC
SNA (°)	0.3	0.994
SNB (°)	0.2	0.997
ANB (°)	0.2	0.997
Wits (mm)	0.3	0.995
Co-Gn (mm)	0.2	0.998
SN-Pal. Pl. (°)	0.2	0.995
SN-GoMe (°)	0.3	0.998
Pal. Pl.-Mand.Pl (°)	0.2	0.998
ArGoMe (°)	0.2	0.997
Pal. Pl.-Occl. Pl. (°)	0.2	0.996
Occl. Pl.-Mand. Pl. (°)	0.2	0.997
I^SN (°)	0.2	0.999
I^Pal. Pl. (°)	0.2	0.999
IMPA (°)	0.2	0.999

No statistically significant differences for any of the cephalometric variables at baseline (Table 3) and at T2 (Table 4) were found between the two groups with the exception of the mandibular unit length (Co-Gn value) that was significantly greater in the pubertal group (*P* = 0.005, and *P* = 0.010, respectively).

**Table 3:** Descriptive statistics and statistical comparisons (independent-samples t tests) of the starting forms (cephalometric values at T1)

Variables	Prepubertal		Pubertal		Diff	*P* value	95% CI of the difference	
	Mean	SD	Mean	SD			Lower	Upper
SNA (°)	78.8	3.5	79.7	3.9	− 0.9	0.404	− 3.2	1.3
SNB (°)	79.0	3.6	79.4	3.2	− 0.4	0.762	− 2.6	1.8
ANB (°)	− 0.3	2.4	0.4	2.4	− 0.7	0.402	− 2.1	0.9
Wits (mm)	− 4.7	2.9	− 4.5	3.2	− 0.2	0.873	− 2.0	1.7
Co-Gn (mm)	98.3	5.1	103.3	5.9	− 5.0	0.005*	− 8.5	− 1.7
SN-Pal. Pl. (°)	7.8	2.8	9.1	4.0	− 1.3	0.220	− 3.4	0.8
SN-GoMe (°)	35.6	6.4	36.9	4.9	− 1.3	0.493	− 4.9	2.4
Pal. Pl.-Mand.Pl (°)	27.8	5.3	27.8	4.7	0.0	0.983	− 3.1	3.2
ArGoMe (°)	131.4	5.9	132.5	5.5	− 1.1	0.537	− 4.7	2.5
Pal. Pl.-Occl. Pl. (°)	9.3	3.3	8.5	3.0	0.8	0.408	− 1.2	2.8
Occl. Pl.-Mand. Pl. (°)	18.6	4.8	19.4	3.2	− 0.8	0.561	− 3.4	1.9
I^SN (°)	105.6	9.6	106.3	6.3	− 0.7	0.775	− 6.0	4.5
I^Pal. Pl. (°)	66.8	9.8	65.2	6.1	1.6	0.554	− 3.7	6.8
IMPA (°)	86.7	8.8	86.1	7.5	0.6	0.834	− 4.6	5.7

The bold indicates the statistical significance

*P < 0.05; *SD* standard deviations; *Diff*. difference; *CI* confidence interval; *Pal.* palatal; *Pl.* plane; *Mand.* mandibular; *Occl.* occlusal

**Table 4:** Descriptive statistics and statistical comparisons (independent-samples t tests) of the final forms (cephalometric values at T2)

Variables	Prepubertal		Pubertal		Diff	*P* value	95% CI of the difference	
	Mean	SD	Mean	SD			Lower	Upper
SNA (°)	80.2	3.2	80.5	3.9	− 0.3	0.803	− 2.5	1.9
SNB (°)	78.6	3.7	79.1	3.4	− 0.5	0.605	− 2.8	1.7
ANB (°)	1.6	2.3	1.4	3.1	0.2	0.865	− 1.5	1.8
Wits (mm)	− 1.6	2.3	− 2.6	4.4	1	0.374	− 1.2	3.0
Co-Gn (mm)	101.9	5.2	106.8	6.5	− 4.9	0.010*	− 8.4	− 1.2
SN-Pal. Pl. (°)	7.5	3.0	8.4	3.3	− 0.9	0.367	− 2.9	1.1
SN-GoMe (°)	35.4	6.9	37.0	6.0	− 1.6	0.424	− 5.7	2.5
Pal. Pl.-Mand.Pl (°)	27.8	5.9	28.6	5.3	− 0.8	0.676	− 4.3	2.8
ArGoMe (°)	130.3	5.7	131.4	5.7	− 1.1	0.560	− 4.6	2.5
Pal. Pl.-Occl. Pl. (°)	9.2	3.1	7.9	3.2	1.3	0.213	− 0.7	3.2
Occl. Pl.-Mand. Pl. (°)	18.7	4.9	20.7	3.6	− 2	0.156	− 4.7	0.8
I^SN (°)	108.0	7.5	105.8	6.3	2.2	0.313	− 2.2	6.6
I^Pal. Pl. (°)	64.5	8.4	66.7	5.9	− 2.2	0.355	− 6.8	2.5
IMPA (°)	83.7	9.2	82.3	7.3	1.4	0.609	− 3.9	6.6

The bold indicates the statistical significance

*P < 0.05; *SD* standard deviations; *Diff*. difference; *CI* confidence interval; *Pal.* palatal; *Pl.* plane; *Mand.* mandibular; *Occl.* occlusal

No statistically significant T1-T2 changes were found between the CS1-2 and CS3-4 groups for any of the angular and linear measurements (Table 5). In both groups the modified SEC III protocol produced favorable increases in SNA and ANB angles and favorable decreases in the SNB angle. The Wits appraisal showed a greater increase in the modified SEC III group treated in CS1-2 (mean: 3.0 mm; SD: 2.2 mm) than in the CS3-4 group (mean: 2.0 mm; SD: 2.7 mm), though this difference was not statistically significant (Table 5).

**Table 5:** Descriptive statistics and statistical comparisons (independent-samples t tests) of the T2-T1 changes

Variables	Prepubertal		Pubertal		Diff	*P* value	95% CI of the difference	
	Mean *Median*	SD *IQR*	Mean *Median*	SD *IQR*			Lower	Upper
SNA (°)	1.5	1.5	0.8	1.6	0.6	0.166	− 0.3	1.7
SNB (°)	− *0.3*	*2.0*	*0.1*	*2.0*	− 0.2	0.295		
ANB (°)	1.8	1.2	1.1	2.1	0.7	0.129	− 0.2	1.8
Wits (mm)	3.0	2.2	2.0	2.7	1.0	0.159	− 0.4	2.6
Co-Gn (mm)	3.7	1.9	3.4	1.7	0.2	0.687	− 0.9	1.4
SN-Pal. Pl. (°)	− 0.2	1.7	− 0.6	1.5	0.3	0.436	− 0.6	1.4
SN-GoMe (°)	− *0.4*	*2.0*	− *0.8*	*2.9*	0.4	0.941		
Pal. Pl.-Mand.Pl (°)	0.0	2.3	0.8	2.0	− 0.8	0.266	− 2.1	0.6
ArGoMe (°)	− 1.1	1.2	− 1.2	1.5	0.1	0.868	− 0.8	0.9
Pal. Pl.-Occl. Pl. (°)	− 0.1	2.8	− 0.5	2.1	0.4	0.593	− 1.2	2.0
Occl. Pl.-Mand. Pl. (°)	0.1	2.6	1.3	2.3	− 1.2	0.124	− 2.8	0.3
I^SN (°)	2.4	8.0	− 0.6	5.6	3.0	0.185	− 1.5	7.4
I^Pal. Pl. (°)	− 2.2	8.7	1.5	3.8	− 3.7	0.096	− 8.1	0.7
IMPA (°)	− 3.0	5.1	− 3.8	4.6	0.8	0.600	− 2.3	3.9

Italics indicates the value for the Median and for the Interquartile Range (IQR)

*SD* standard deviations; *IQR* Interquartile range; *Diff*. difference; *CI* confidence interval; *Pal.* palatal; *Pl.* plane; *Mand.* mandibular; *Occl.* occlusal

It should be noted that no temporomandibular disorders were reported before and during treatment in the two groups. Moreover, no onset of tooth decay was reported for any patients included in the study during the modified SEC III treatment duration.

## Discussion

This retrospective study was performed to detect the optimal timing for the treatment of Class III malocclusion with the modified SEC III treatment in patients with mixed dentition divided by cervical vertebral maturation (CS1-2 versus CS3-4). It is widely accepted that early treatment has manifold reasons to be performed as soon as a Class III malocclusion is diagnosed in young patients [2]. In any case, clarifications in this regard were investigated in several clinical studies using other protocols to treat similar types of patients with Class III malocclusion with different ages [4, 5] and skeletal maturation assessed through the cervical vertebral maturation method [6], as in this study. At this regard, recent studies demonstrated that this method can differentiate between prepeak (CS1-2) and peak groups (CS3-4) without any influence by secular trends regarding duration of the skeletal maturation in relation to peak height velocity as can occur using age [33–36]. The use of maxillary expander and facial mask in the RME/FM protocol have shown that a larger maxillary expansion and protraction may occur in early mixed dentition due to a favorable sutural response. It was found that the optimal timing to start treatment of the Class III disharmony with this protocol was in the early mixed dentition (or late deciduous dentition) [6].

The findings of the current study mostly agree with the data reported by Perillo et al. [16] who found an increase in the SNA angle, a reduction in SNB angle, and an improvement of ANB angle and Wits appraisal after SEC III treatment in prepubertal patients comparable with CS1-2 group of this study and with the RME/FM protocol when used before puberty. The comparison between CS1-2 and CS3-4 groups confirmed favorable increases in the skeletal sagittal variables in both groups with improvements in SNA, ANB and in the Wits appraisal that were greater (though not statistically significant) in the prepubertal group.

As for the vertical pattern, favorable reduction of the SN-Mandibular Plane was detected in both groups after treatment with no significant differences.

A favorable control of the vertical skeletal relationships produced by the modified SEC III protocol seems to be related both to the use of chincup in association with anterior elastics and to the single contact point of the bonded expander with a lower splint at the level of the last molars creating a wedge effect [19].

These outcomes confirmed that early treatment of Class III malocclusion with the modified SEC III protocol offers several advantages especially in children with hyperdivergent growth pattern if a good to moderate compliance with the removable appliances included in the protocol is present [22]. A limitation of the present study was that patients’ compliance was obtained from the clinical chart after interviewing each patient and also his/her parents at each appointment. Nowadays objective methods to assess patients’ compliance with removable appliances are available (sensors) and they should be used increasingly in clinical studies [37, 38].

Differently from the use of RME/FM protocol, the modified SEC III protocol seemed to be equally effective regardless of the age, dentition stage, or skeletal maturation of the children treated [6, 22].

A control group was not considered in this study because, in previous studies, the SEC III protocol was already compared to an untreated group of growing patients with Class III malocclusion and short-term outcomes showed favorable both maxillary and mandibular skeletal changes [16].

Some limitations of this study were the short-term evaluation and its retrospective nature with relatively small sample size. Another limitation was that skeletal maturity was assessed with the cervical vertebral maturation, a method whose reliability remains controversial in the literature. Recent systematic reviews, however, found that the CVM method might be considered as reliable in skeletal maturation assessment as the hand-wrist method in growing subjects [39].

Therefore, further long-term, prospective studies on larger samples are needed to assess the stability of the dentoskeletal effects produced in prepubertal and pubertal growth stages.

## Conclusions

The treatment of Class III dentoskeletal disharmony with the modified SEC III protocol produced similar sagittal and vertical dentoskeletal effects in prepubertal and pubertal patients. Thus, this treatment protocol produced favorable changes in growing subjects regardless of the cervical vertebral maturation stages from CS1 to CS4.

## Data Availability

The datasets used and/or analysed during the current study are available from the corresponding author on reasonable request.

## Abbreviations

CS: Cervical vertebral maturation stage
SEC: Splints, class III elastics and chincup
ANB: Point A-Nasion-point B
MME: Method of moments estimator
ICC: Intraclass correlation coefficients
SD: Standard deviation
Co: Condylion
Gn: Gnation
SNA: Sella-Nasion-point A angle
SNB: Sella-Nasion-point B angle
RME/FM: Rapid maxillary expander/face mask
